# The early effects of external and internal strategies on working memory updating training

**DOI:** 10.1038/s41598-018-22396-5

**Published:** 2018-03-06

**Authors:** Matti Laine, Daniel Fellman, Otto Waris, Thomas J. Nyman

**Affiliations:** 1 Department of Psychology, Åbo Akademi University, Turku, Finland; 2 Turku Brain and Mind Center, University of Turku, Turku, Finland

## Abstract

The mechanisms underlying working memory training remain unclear, but one possibility is that the typically limited transfer effects of this training reflect adoption of successful task-specific strategies. Our pre-registered randomized controlled trial (N = 116) studied the early effects of externally given vs. internally generated strategies in an updating task (n-back) over a 5-day period with a single 30-minute training session. Three groups were employed: n-back training with strategy instruction (n = 40), n-back training without strategy instruction (n = 37), and passive controls (n = 39). We found that both external and internal strategy use was associated with significantly higher posttest performance on the trained n-back task, and that training with n-back strategy instruction yielded positive transfer on untrained n-back tasks, resembling the transfer pattern typically seen after the ordinary uninstructed 4–6-week working memory training. In the uninstructed participants, the level of detail and type of internally generated n-back strategies at posttest was significantly related to their posttest n-back performance. Our results support the view that adoption of task-specific strategies plays an important role in working memory training outcomes, and that strategy-based effects are apparent right at the start of training.

## Introduction

Working memory (WM) training has stirred considerable research and public interest. This training approach deviates strongly from traditional mnemonic techniques, as it involves repeated adaptive practice with WM tasks. The basic idea is that intensive continuous challenges close to maximum manageable difficulty level elicit plastic changes in the brain that increase the capacity of the targeted memory system^1^. This has been coined as the Capacity hypothesis of WM training^2^, according to which training expands the mental platform that WM represents.

The initial WM training outcomes were very promising by suggesting transfer even to other cognitive domains such as fluid intelligence^3^, mathematics^4^, and reading comprehension^5^. These far transfer effects were thought to reflect the key role that WM plays as the mental platform of ongoing cognitive activities. However, after a decade of research and several meta-analyses^6,7^, it seems that positive transfer from working memory training is limited to untrained WM tasks only. Furthermore, the most recent meta-analysis on a popular WM training approach, namely training with adaptive n-back tasks, found that more substantial near transfer effects are even more limited, involving only WM tasks that are structurally similar to the trained tasks^8^. Such a narrow, task-specific near transfer could be accounted for by strategies suited for the trained tasks that the participants have developed during training. Accordingly, it has been suggested that strategy influences rather than increased capacity may underlie WM training outcomes^7–9^. This competing view has been labeled as the Strategy Mediation hypothesis^2^, and the present study sought to examine the viability of this theoretical claim.

A limited literature exists on strategy training and strategy use with WM tasks, albeit the evidence is mostly from a single paradigm, namely from complex span tasks. In a strategy training study by McNamara and Scott^10^, practicing with a semantic strategy (generating a story based on a list of unrelated words) over four sessions improved word recall both on a word list task and a complex span task. This finding has been confirmed in subsequent studies. Turley-Ames and Whitfield^11^ found that for low WM span individuals, strategy instruction (especially a rehearsal strategy that was contrasted with an imagery strategy and a semantic strategy) improved complex span performance. Carretti and colleagues reported that imagery strategy instruction for word lists improved performance on a subsequent word list recall task, and also on a WM task requiring semantic categorization of to-be-remembered words^12^. Furthermore, Bailey and colleagues^13^ reported performance improvement on their complex span task after strategy practice with word list learning, albeit paired-associate cued recall and a memory-based shape selection task did not show training benefits.

Besides practice with an externally provided strategy, also the use of internally generated strategies as measured by self-reports has been found to be related to better complex span performance in several studies^9,10,14–16^. Of particular relevance to WM training research in adults is the study by Dunning and Holmes^9^ who gathered participants’ strategy reports at pre- and posttest in a randomized controlled trial with adaptive WM training. On all but one test, a significant increase in the self-reported use of a grouping strategy at posttest was observed for the adaptive training group as compared to active and passive controls. Increases in performance gains and grouping strategy use co-occurred on two of the four pre-post tasks. These two tasks shared both the task structure and stimulus materials with the Cogmed program used for training. Thus, these results mainly indicate increased use of internal strategies on the same tasks that had been trained.

The present study tested the Strategy Mediation hypothesis by examining the relationships between WM strategy use (internal, external) and WM training outcomes in a randomized controlled trial. We employed short (single-session) training as there is evidence for significant performance improvements on WM tasks already within the first two test sessions and even within a single session^17^. This could suggest not only the consolidation of stimulus-response mappings, but possibly also an early onset for strategy-based effects. Moreover, as previous relevant studies are mostly limited to a single WM paradigm, namely complex span, we chose to study strategy-based effects on n-back that has been a popular WM updating paradigm in both basic WM research and as a training task. Our hypotheses were as follows. If the Strategy Mediation hypothesis holds, (a) an externally provided n-back strategy should improve performance on the n-back training task and on untrained n-back tasks that should benefit from the same strategy, but not on other untrained WM tasks; (b) the use of internally generated strategies should also be related to higher WM performance at posttest.

## Results

Background and pretest characteristics. Statistical comparisons by one-way ANOVAs and a Chi-square indicated that the three groups did not differ on age, education, gender distribution, or pretest n-back performance (Table 1).

**Table 1:** Background and pretest characteristics of the three groups.

	Strategy training group	Active control group	Passive control group	*p*
*n*	40	37	39	
Years of Education	14.88 (3.88)	14.97 (3.93)	14.74 (3.42)	0.97
Age (Years)	24.32 (4.63)	24.11 (4.01)	24.08 (4.49)	0.96
Gender F/M	29/11	28/9	28/11	0.92
Pretest n-back composite	0.24 (5.15)	−0.58 (3.73)	0.30 (6.09)	0.71

Values in parentheses are standard deviations. *p* values are calculated from one-way ANOVAs for continuous variables and χ^2^ test for the categorical variable gender.

The n-back composite score comprised of the summed values of the z-transformations of the average and maximum level accuracy in the adaptive digit n-back task, and the d-prime values and RTs on correct target responses in the letter n-back and color n-back tasks.

### Training session data

**Figure 1:**
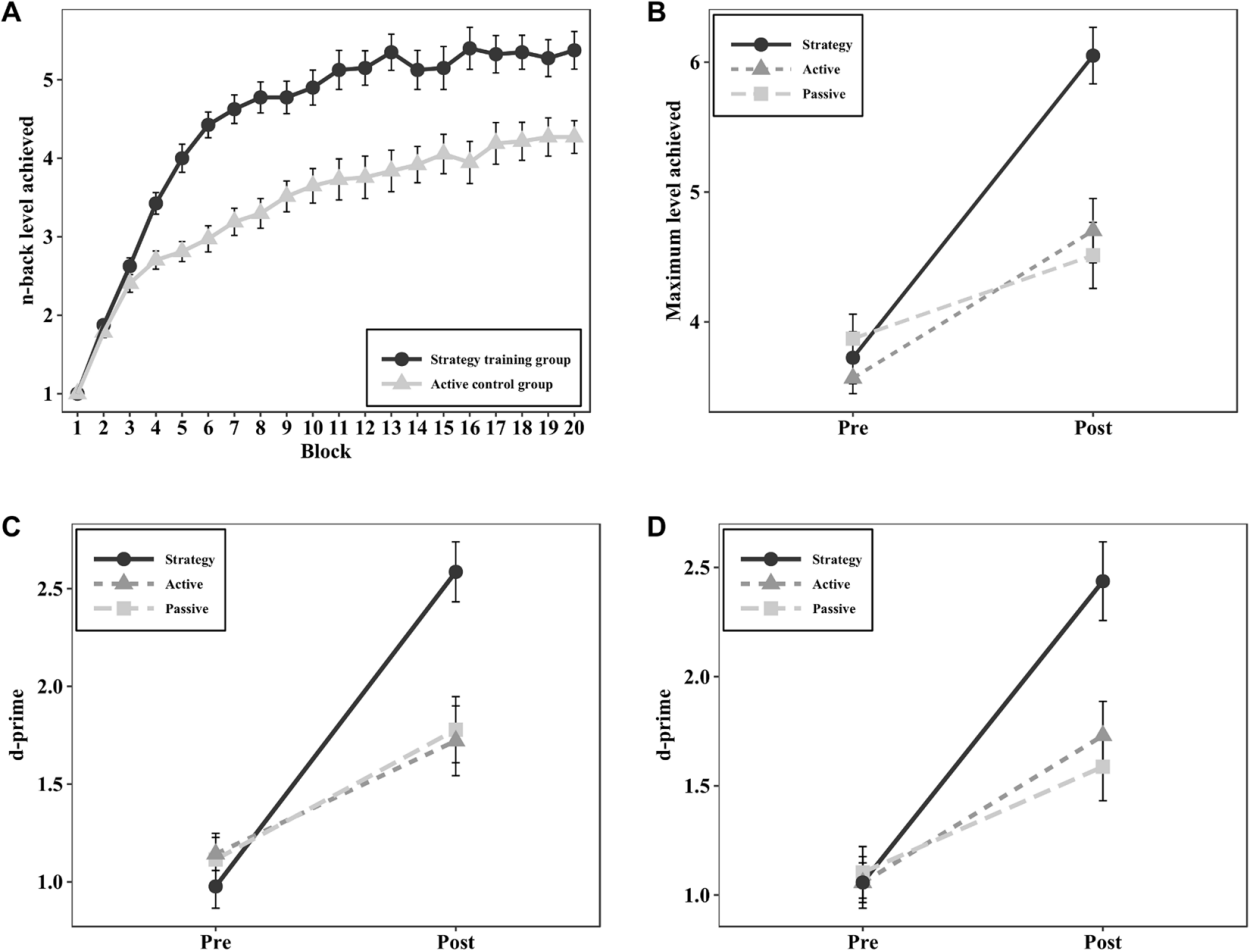
(**A**) Training data across the 20 training blocks. The *Y*-axis represents the n*-*back level reached. Error bars represent standard error of means. The strategy training group reached a higher n-back level already on the fourth training block (*p* < 0.001). **(B)** Pre- and posttest performance in the trained n-back task with maximum n*-*back level achieved as the outcome variable. The strategy training group showed a significant improvement in the trained task as compared to the active control group (*p* < 0.001) and the passive control group (*p* < 0.001). **(C)** shows pre- and posttest performance in the 3-back task with letters, and **(D)** depicts the corresponding results for the 3-back task with colors. In both tasks, the dependent variable was d-prime. Both untrained 3-back tasks showed significant improvement in the strategy training group, compared with the active control group (*p* = 0.001) and the passive control group (*p* < 0.001).

Figure 1A shows the improvement over the 20 n-back blocks during the 30-minute training session for the strategy training group and the active control group. The achieved n-back level started to deviate between groups after the first few blocks. An independent samples t-test confirmed that the strategy training group reached a higher n-back level already on the fourth training block compared to the active control group, *t*(75) = 3.98, *p* < 0.001.

### Alertness, motivation, and expectations

Expectations of improvement during the training session were assessed a moment before the training session started. The expectations of the strategy training group were higher than those of the active control group, *t*(75) = 2.91, *p* = 0.005. This difference most likely reflects the fact that the instructed strategy had been introduced to the strategy training group right before evaluating the improvement expectations. For all groups, we also examined the improvement expectations on individual tasks from pretest to posttest. The instructed strategy had been introduced to the strategy training group before responding to this expectation survey. One-way ANOVAs revealed no significant group differences in the n-back task with letters, *F*(2, 113) = 1.75, *p* = 0.179, n-back task with colors, *F*(2, 113) = 1.73, *p* = 0.182, Selective updating of digits, *F*(2, 113) = 2.81, *p* = 0.064, Forward digit span *F* < 1, or in Running memory, *F* < 1. In the trained digit n-back task there was, however, a significant effect of group, *F*(2, 113) = 4.62, *p* = 0.012. A closer examination of the improvement expectations in the trained digit n-back task using orthogonal contrasts showed no difference between the two intervention groups, *t*(75) = −1.93, *p* = 0.056. However, there was a significant group difference between the strategy training group and the passive control group, *t*(77) = −2.99, *p* = 0.003, indicating that the strategy training group expected to improve more on the trained digit n-back task from pre- to posttest compared to the passive control group. The expectations of the active control group did not differ significantly from the expectations of the passive control group in relation to the trained digit n-back task, *t*(74) = −1.02, *p* = 0.311.

We also examined the self-reported levels of alertness and motivation between the two training groups immediately after the participants had completed the training session. Independent samples t-test revealed no group differences in either motivation, *t*(75) = 1.10, *p* = n.s., or alertness, *t* < 1.

### Pre- and posttest measures

Participants scoring three times the interquartile range above or below the 1st or the 3rd quartile at pretest were considered as univariate outliers and were excluded from the analyses in question. These univariate outliers are reported separately for each measure. The pre-post values (means, standard deviations, pre-post correlations, and effect sizes) per group are shown in Table 2, and ANCOVA statistics are presented in Table 3.

**Table 2:** Mean values (standard deviations) for the pre-post measures per group at pre- and posttest.

	Strategy training group (n = 40)^c^				Active control group (n = 37)^a,c^				Passive control group (n = 39)^b,c^			
	Pre	Post	*r*	*d*	Pre	Post	*r*	*d*	Pre	Post	*r*	*d*
***Trained n-back with digits***												
Maximum level	3.73 (1.26)	6.05 (1.38)	0.45	1.76	3.57 (0.73)	4.70 (1.51)	0.41	0.89	3.87 (1.17)	4.51 (1.59)	0.63	0.44
Average level	2.44 (0.74)	3.80 (0.92)	0.39	1.61	2.38 (0.54)	3.21 (0.91)	0.33	1.08	2.61 (0.82)	3.01 (0.96)	0.74	0.44
***Task-specific near transfer***												
2-back with letters (d-prime)	2.36 (0.82)	3.04 (0.98)	0.34	0.75	2.08 (0.70)	2.81 (0.61)	0.09	1.11	2.41 (0.81)	2.68 (0.79)	0.46	0.34
3-back with letters (d-prime)	0.98 (0.71)	2.58 (0.97)	0.45	2.07	1.14 (0.51)	1.72 (1.08)	0.14	0.67	1.11 (0.82)	1.78 (1.04)	0.62	0.69
2-back with colors (d-prime)	2.35 (0.77)	2.87 (1.29)	0.56	0.45	2.33 (0.82)	2.75 (0.82)	0.35	0.50	2.46 (0.86)	2.82 (0.88)	0.54	0.42
3-back with colors (d-prime)	1.06 (0.74)	2.44 (1.12)	0.43	1.41	1.06 (0.54)	1.73 (0.94)	0.24	0.83	1.10 (0.74)	1.59 (0.98)	0.56	0.55
2-back with letters (RT in msec)	873 (145)	695 (156)	0.38	1.18	849 (121)	720 (114)	0.62	1.09	845 (137)	710 (124)	0.49	1.07
3-back with letters (RT in msec)	918 (169)	702 (156)	0.41	1.33	897 (117)	747 (151)	0.39	1.10	858 (144)	747 (138)	0.54	0.78
2-back with colors (RT in msec)	848 (148)	719 (167)	0.60	0.81	872 (149)	724 (123)	0.71	1.07	829 (131)	714 (130)	0.57	0.88
3-back with colors (RT in msec)	903 (167)	737 (185)	0.67	0.94	917 (146)	785 (150)	0.59	0.90	854 (162)	790 (157)	0.56	0.40
***Task-general near transfer (% accuracy)***												
Selective updating of digits	59.70 (15.07)	65.10 (14.80)	0.66	0.36	54.70 (11.04)	60.65 (12.69)	0.53	0.49	57.59 (13.27)	63.90 (13.51)	0.82	0.47
Forward digit span (correct items)	62.50 (17.19)	67.30 (17.58)	0.57	0.28	59.85 (18.01)	61.39 (15.23)	0.54	0.09	61.33 (16.47)	63.32 (19.73)	0.70	0.11
Forward digit span (maximum span)	61.25 (22.44)	67.50 (16.13)	0.39	0.32	56.49 (21.76)	59.46 (17.15)	0.72	0.15	61.79 (17.60)	63.59 (18.42)	0.51	0.10
Running memory	74.84 (20.23)	80.23 (16.03)	0.58	0.29	74.66 (16.43)	82.18 (12.28)	0.46	0.51	72.52 (16.85)	80.37 (16.28)	0.65	0.47

Values in parentheses are standard deviations. *r* = correlation between pre- and posttest.

Cohen’s *d* represents effect sizes for correlated samples.

^a^One extreme outlier in the active control group in 2-back with letters (both d-prime and RT) (*n* = 36).

^b^One extreme outlier in the passive control group in 3-back with letters (d-prime) (*n* = 38).

^c^Two participants with verified color blindness were excluded from the color n-back task (strategy group *n* = 39, active control group *n* = 36).

**Table 3:** ANCOVA results with planned contrasts for the trained task and for the transfer measures.

	Condition			Strategy training group vs Active controls				Strategy training group vs Passive controls			
	*F*	*p*	${n}_{p}^{2}$	*t*	*p*	*d*	95% CI	*t*	*p*	*d*	95% CI
***Trained n-back with digits***											
Maximum level	**17.09**	**<0** **.001**	**0.23**	**−4.18**	**<0** **.001**	**−**0.95	**−**1.43, **−**0.48	**−5.60**	**<0** **.001**	**−**1.26	**−**1.74, **−**0.78
Average level	**12.58**	**<0** **.001**	**0.18**	**−2.97**	**0.013**	**−**0.68	**−**1.14, **−**0.22	**−4.97**	**<0** **.001**	**−**1.12	**−**1.60, **−**0.65
***Task-specific near transfer***											
2-back with letters (d-prime)	2.43	0.195	0.04	**−**0.76	0.632	**−**0.17	**−**0.63, 0.28	**−**2.18	0.082	**−**0.49	**−**0.94, **−**0.04
3-back with letters (d-prime)	**12.83**	**<0** **.001**	**0.19**	**−4.52**	**<0** **.001**	**−**1.03	**−**1.51, **−**0.56	**−4.21**	**<0** **.001**	**−**0.95	**−**1.42, **−**0.49
2-back with colors (d-prime)	0.19	0.872	0.00	**−**0.49	0.794	**−**0.11	**−**0.57, 0.34	**−**0.55	0.763	**−**0.13	**−**0.57, 0.32
3-back with colors (d-prime)	**9.85**	**<0** **.001**	**0.15**	**−3.31**	**0** **.005**	**−**0.76	**−**1.23, **−**0.30	**−4.20**	**<0** **.001**	**−**0.95	**−**1.42, **−**0.48
2-back with letters (RT)	1.02	0.545	0.02	1.35	0.348	0.31	**−**0.14, 0.76	1.07	0.462	0.24	**−**0.20, 0.68
3-back with letters (RT)	3.07	0.124	0.05	1.79	0.348	0.41	**−**0.04, 0.86	2.37	0.058	0.54	0.09, 0.99
2-back with colors (RT)	0.21	0.872	0.00	**−**0.42	0.800	**−**0.10	**−**0.55, 0.36	0.23	0.872	0.05	**−**0.39, 0.50
3-back with colors (RT)	3.99	0.060	0.07	1.29	0.348	0.30	**−**0.16, 0.75	2.82	0.058	0.64	0.19, 1.10
***Task-general near transfer***											
Selective updating of digits	0.15	0.882	0.00	**−**0.41	0.800	**−**0.09	**−**0.54, 0.35	0.12	0.904	0.03	**−**0.41, 0.47
Forward digit span (correct items	0.96	0.557	0.02	**−**1.32	0.348	**−**0.30	**−**0.75, 0.15	**−**1.02	0.481	**−**0.23	**−**0.67, 0.21
Forward digit span (maximum span)	1.66	0.348	0.03	**−**1.76	0.178	**−**0.40	**−**0.86, 0.05	**−**1.26	0.353	**−**0.28	**−**0.73, 0.16
Running memory	0.27	0.871	0.00	0.72	0.641	0.16	**−**0.28, 0.61	0.45	0.800	0.10	**−**0.35, 0.55

Significant effects are bolded (*p*-values are corrected using the Benjamini-Hochberg procedure for multiple comparisons). Planned (orthogonal) contrasts are reported for all outcomes in this table.

Note. Cohen’s *d* is computed from estimated posttest measurement scores adjusted for pre-measurements in the ANCOVA.

95% CI represents confidence intervals around *d*.

#### The trained n-back task with digits

**Figure 2:**
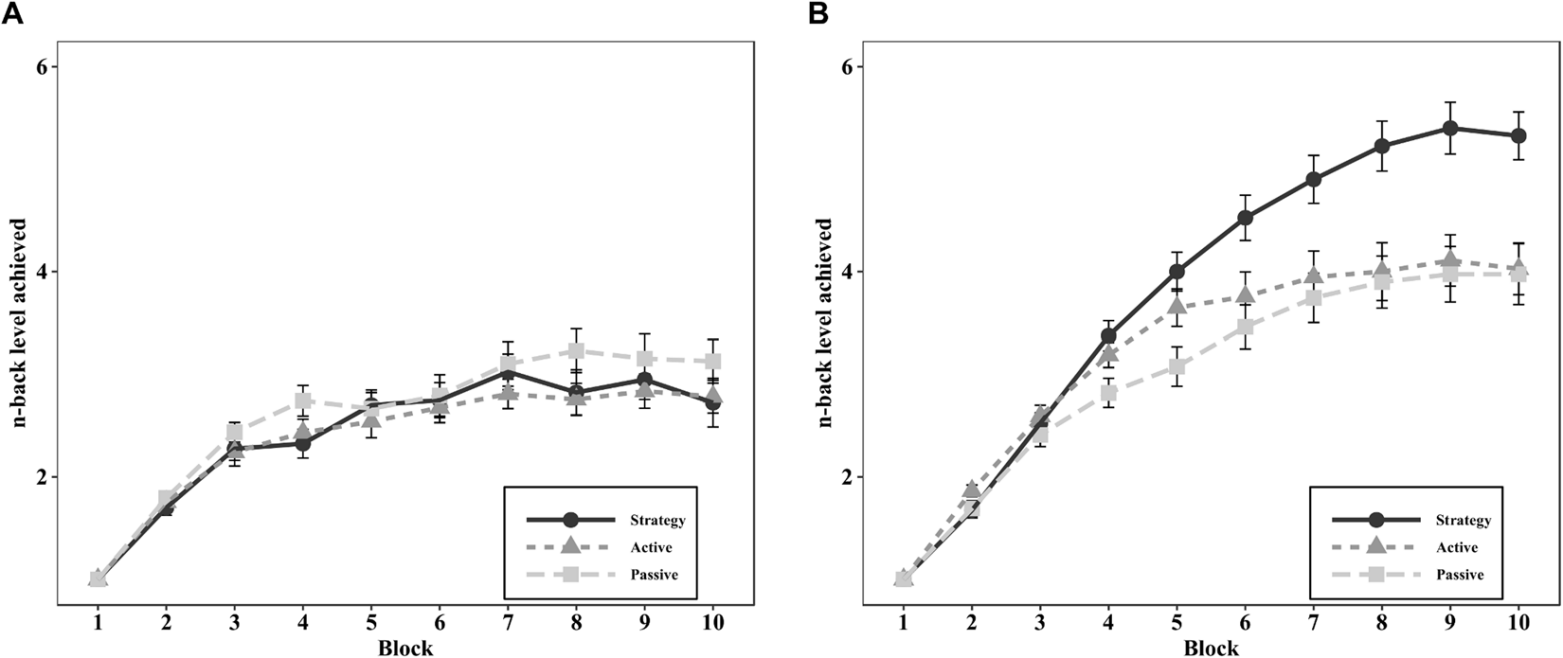
Average performance across the 10 blocks of the trained n-back task in the three groups at **(A)** pretest and **(B)** posttest. The *Y*-axis represents the n-back level reached. Error bars represent standard error of means.

In the trained adaptive n-back task with digits, we used two different outcome measures, namely maximum and average n-back level obtained during the 10 trials. No data were excluded from these analyses. For the maximum n-back level outcome measure, ANCOVA revealed a significant main effect of group, *F*(2, 112) = 17.09, *p* < 0.001, ${\eta }_{p}^{2}$ = 0.23. Planned contrasts showed that the adjusted posttest means of the strategy training group were significantly higher compared to the active control group, *t*(75) = −4.18, *p* < 0.001, *d* = −0.95, 95% CI [−1.43, −0.48], and the passive control group, *t*(77) = −5.60, *p* < 0.001, *d* = −1.26, 95% CI [−1.74, −0.78] (see Fig. 1B). In line with these results, ANCOVA on the average n-back level revealed a main effect of group *F*(2, 112) = 12.58, *p* < 0.001, ${\eta }_{p}^{2}$ = 0.18. Accordingly, planned contrasts showed that the strategy training group obtained a higher adjusted average n-back level at posttest compared with the active control group, *t*(75) = −2.97, *p* = 0.013, *d* = −0.68, 95% CI [−1.14, −0.22], as well as with the passive control group, *t*(77) = −4.97, *p* < 0.001, *d* = −1.12, 95% CI [−1.60, −0.65]. Block-by-block performance on the trained digit n-back task at pre- and posttest is depicted in Fig. 2.

#### The untrained n-back task with letters

One active control group participant was removed for being an extreme outlier on 2-back pretest accuracy. Another single univariate outlier from the passive control group was removed from the 3-back pretest accuracy analysis. ANCOVA on d-prime in the 2-back task showed no significant main effect of group, *F*(2, 111) = 2.43, *p* = 0.195, ${\eta }_{p}^{2}$ = 0.04, but in the more demanding 3-back condition, the main effect of group was highly significant, *F*(2, 111) = 12.83, *p* < 0.001, ${\eta }_{p}^{2}$ = 0.19. Planned contrasts showed that the adjusted posttest mean of the strategy training group was significantly higher compared to those of the active control group, *t*(75) = −4.52, *p* < 0.001, *d* = −1.03, 95% CI [−1.51, −0.56] and the passive control group, t(76) = −4.20, *p* < .001, *d* = −0.95, 95% CI [−1.42, −0.49] (see Fig. 1C).

As regards the RT analyses, one active control group participant was excluded for being an extreme outlier at pretest 2-back with letters. No participants were excluded in the 3-back RT analyses. ANCOVAs on the RTs showed no main effect of group in either the 2-back task, *F*(2, 111) = 1.02, *p* = 0.545, ${\eta }_{p}^{2}$ = 0.02, or the 3-back task, *F*(2, 112) = 3.07, *p* = 0.124, ${\eta }_{p}^{2}$ = 0.05.

#### The untrained n-back task with colors

One strategy group participant and one active control group participant reported red-green color blindness that was also confirmed by the Ishihara color test at pretest. Only these participants were excluded when analyzing the color n-back task.

ANCOVA on d-prime did not show a significant main effect of group for the 2-back, *F* $$ < 1$$, but the group difference on the more demanding 3-back task was highly significant, *F*(2, 110) = 9.85, *p* < 0.001, $${\eta }_{p}^{2}$$ = 0.15. Further examination with planned contrasts showed that the strategy training group obtained a significantly higher adjusted posttest mean score than the active control group, *t*(73) = −3.31, *p* = 0.005, *d* = −0.76, 95% CI [−1.23, −0.30] and the passive control group, *t*(76) = −4.20, *p* < 0.001, *d* = −0.95, 95% CI [−1.42, −0.48] (see Fig. 1D).

Regarding the RT analyses, ANCOVA showed no statistically significant main effect of group in the 2-back task, *F* $$ < 1$$, or in the 3-back task, *F*(2, 110) = 3.99, *p* = 0.060, $${\eta }_{p}^{2}$$ = 0.07.

#### Task-general near transfer

No univariate extreme outliers were identified on the task-general near transfer tasks. ANCOVAs showed no significant main effects of group in the Selective updating of digits, *F* $$ < 1$$, Forward digit span with correctly recalled digits, *F*  $$ < 1$$, Forward digit span with maximum span, *F*(2, 112) = 1.66, *p* = 0.348, $${\eta }_{p}^{2}$$ = 0.03, or Running memory, *F*  $$ < 1$$. More detailed ANCOVA statistics are presented in Table 3.

### The relationship between self-generated n-back strategies and n-back task performance in the controls at posttest

As the strategy reports for the strategy training group showed that already during the 30-minute training session 34 out of the 37 participants had used the instructed strategy, we ran post hoc analyses on the associations between reported strategy use and n-back performance at posttest only for the controls. Thus, the present analyses examined whether the level of detail and type of controls’ self-reported n-back strategy use at posttest was related to posttest performance on the same tasks.

#### N-back task performance as a function of the level of detail of the self-generated strategy

We conducted a linear regression analysis to probe whether the level of detail in the reported use of strategies in the three n-back tasks at posttest had some predictive value for the corresponding posttest n-back performance. Both the n-back strategy variable and the n-back performance variable summed up the scores over the three n-back tasks. The n-back posttest composite score comprised of the summed values of the z-transformations of the posttest average and maximum level reached in the trained digit n-back task, and the posttest d-prime variables in the 3-back tasks with letters and with colors. We summed up all three n-back tasks due to the limited scale of the level of detail in strategy (range 1 to 3 per n-back task; see below).

The scoring procedure for the self-reported freely described n-back strategies was based on the level of detail in the strategy descriptions for each of the three n-back tasks at posttest. Two independent evaluators conducted the scoring, and discrepancies were solved afterwards through consensus. Reports of no strategy use were scored as zero points. Report of a vague, non-specific strategy (e.g., “I memorized the digits in my mind”; “I used intuition”) gave one point, and report of a clear strategy with at most one detail (e.g., “I repeated the letters in my mind in two or three groups”; “I memorized the digits in pairs, such as 52–48”) received two points. Lastly, three points were given if the participant described a clear strategy together with two or more details (e.g., “I split the digits into different series, and compared those to each other”; “I tried to keep n-numbers of a letter series in mind, and change it when the letters changed”).

This scoring procedure was applied to all three n-back tasks at posttest, yielding a summative score that ranged from 0 to 9. Inter-rater reliability analysis (two raters; a linearly weighted kappa analysis, κ_w_)^18^ applied to our ordinal scale data revealed a very good reliability for the scoring procedure, κ_w_ = 0.844, 95% CI [0.774, 0.914]^19^.

**Figure 3:**
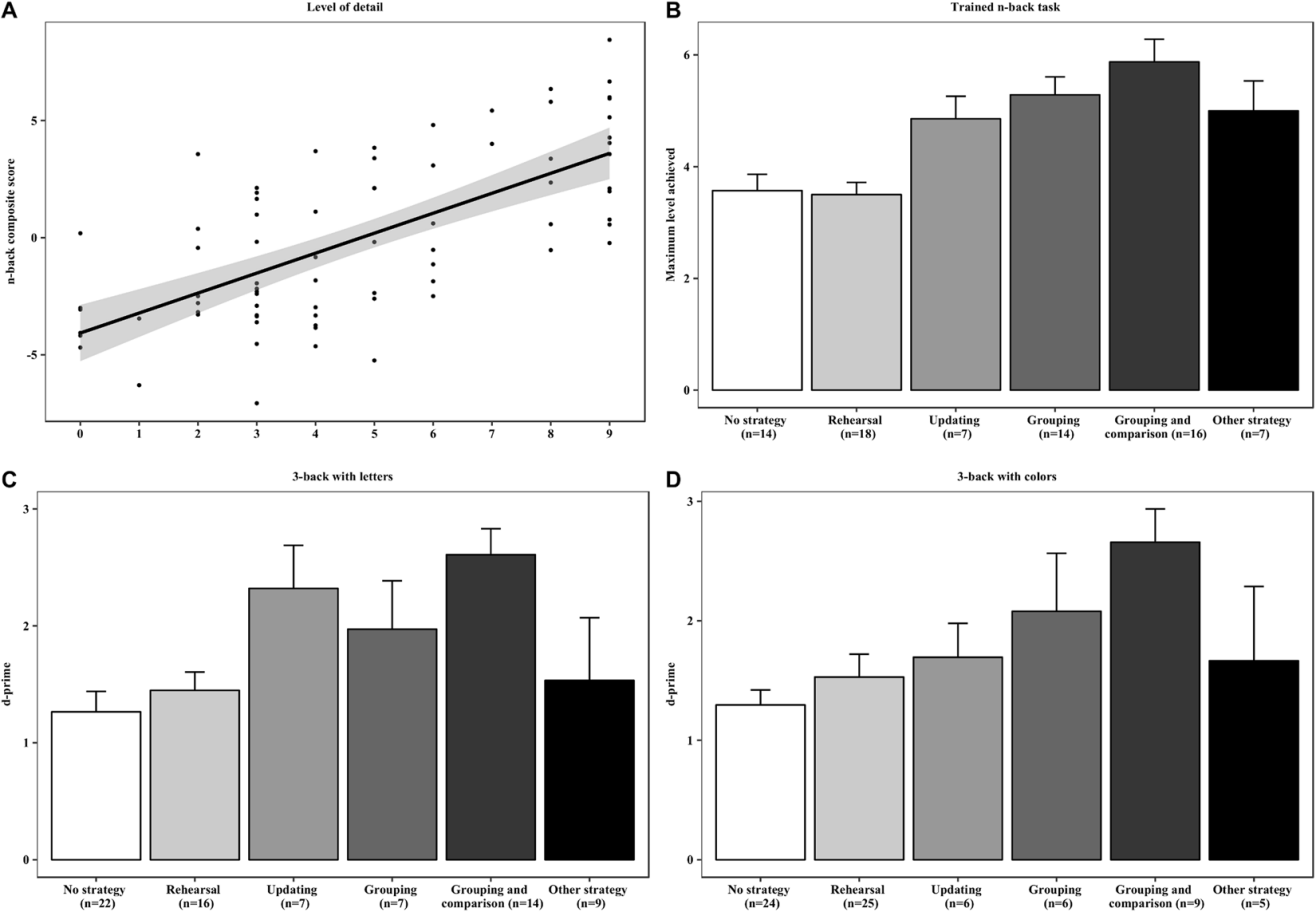
(**A**) Level of detail in strategy report and overall n-back performance at posttest. The panel shows a regression plot with the level of detail in strategy report as the predictor (X-axis) and the n-back composite score (Y-axis) as the dependent variable. The n-back composite sums up the posttest z-values of the average and maximum n-back level reached in the trained digit n-back task, and the d-prime values in the untrained letter and color 3-back tasks. Grey shaded regions represent 95% confidence intervals on the slope. (**B**) Strategy type and performance in the trained n-back task at posttest. (**C**) Strategy type and performance in the untrained letter n-back task at posttest. (**D**) Strategy type and performance in the untrained color n-back task at posttest. Note. All plots and variables are based on the posttest data from the two control groups (i.e., active and passive controls combined). The whiskers in 2B, 2C, and 2D represent standard error of means.

The role of spontaneous strategy use on n-back performance was studied with a regression analysis in all control participants except the one suffering from color-blindness. Thus, the final sample of controls used here included 75 participants. The analysis indicated that the level of detail of reported strategies explained 45.6% of the variance in the n-back composite score at posttest, (R^2^ = 0.456, *β*  = 0.675, *t*(75) = 7.83, *p* < 0.001) (see Fig. 3A).

#### N-back task performance as a function of the type of the self-generated strategy

In order to investigate further the role of self-generated strategies in n-back performance at posttest, we classified the control participants’ strategy descriptions in the n-back tasks at posttest according to their type. The classification scheme included the following categories based on a recent WM strategy analysis by Morrison and colleagues^20^ and a preliminary inspection of the present strategy reports: Rehearsal, Grouping, Updating, Grouping and comparison, Semantics, Phonology, Imagery, Familiarity, Guessing, Other strategy, or No strategy use (see Supplementary Table S1). Two raters classified the strategy reports independently and solved discrepancies afterwards through consensus. An unweighted Cohen’s kappa inter-rater reliability for the two independent raters showed an overall good level of agreement in each n-back task (trained digit n-back, κ = 0.708, 95% CI [0.594, 0.822]; letter n-back, κ = 0.656, 95% CI [0.538, 0.775]; color n-back, κ = 0.693, 95% CI [0.577, 0.810])^19^.

The distribution of strategy types employed in the three n-back tasks at posttest can be found in Supplementary Table S2. Strategies with less than 3% of reports (Semantics, Phonology, Imagery, Familiarity, and Guessing) were included in the Other strategy category. Thus, the final list comprised of six categories, namely No strategy, Rehearsal, Updating, Grouping, Grouping and Comparison, as well as Other strategy. No strategy served as the baseline when we examined with one-way ANOVAs whether use of a specific strategy type was related to a higher performance on a given n-back task. In each model, the dependent variable was n-back posttest performance (maximum level achieved for the trained adaptive digit n-back task, d-primes for the untrained letter and color 3-back tasks), and strategy type served as the between-subjects factor. Before running the analyses, we excluded the same cases as in the pre-posttest analyses (i.e., the univariate outlier in the letter 3-back and the participant with color-blindness in the color 3-back). A significant main effect of strategy type was followed up by contrasting the n-back performance of the participants not reporting any strategy against those reporting either Rehearsal, Updating, Grouping, Grouping and comparison, or Other strategy.

Figure 3 panels B, C, and D, summarize the n-back posttest performance as a function of strategy type at posttest, separately for each n-back task. ANOVA on the trained digit n-back task showed a significant main effect of strategy type, *F*(5, 70) = 9.23, *p* < 0.001, ${\eta }_{p}^{2}$ = 0.40. Participants using either Updating, *t*(19) = 2.24, *p* = 0.003, *d* = 1.19, Grouping, *t*(26) = 3.66, *p* < 0.001, *d* = 1.49, Grouping and comparison, *t*(28) = 5.08, *p* < 0.001, *d* = 1.64, or Other strategy, *t*(21) = 2.49, *p* < 0.001, *d* = 1.19, performed significantly better as compared to those not reporting any strategies. However, the n-back performance did not differ between the No strategy group and the Rehearsal group, *t* < 1. ANOVA on the letter 3-back revealed also a significant main effect of strategy type, *F*(5, 69) = 4.40, *p* < 0.001, ${\eta }_{p}^{2}$ = 0.24, and pairwise comparisons showed that those using Updating, *t*(27) = 2.55, *p* = 0.013, *d* = 1.23, or Grouping and comparison, *t*(34) = 5.08, *p* < 0.001, *d* = 1.64, performed significantly better at posttest compared with the No strategy group. WM performance did not differ among those using Rehearsal, *t* < 1, Grouping, *t*(27) = 1.71, *p* = 0.092, *d* = 0.80, or Other strategies, *t* < 1, as compared with the No strategy group. Also ANOVA on the color 3-back revealed a significant main effect, *F*(5, 69) = 3.50, *p* = 0.007, ${\eta }_{p}^{2}$ = 0.20. Here, only the participants using Grouping and comparison were significantly more accurate than the No strategy group, *t*(31) = 3.95, *p* < 0.001, *d* = 2.00, whereas the participants using Rehearsal, *t* < 1, Updating, *t* < 1, Grouping, *t*(28) = 1.95, *p* = 0.056, *d* = 1.04, or Other strategies, *t* < 1, did not differ from those not reporting any strategy.

## Discussion

The present study set out to test the Strategy Mediation hypothesis of WM training by examining the effects of external and internal strategy use in single-session WM training with an n-back task. The results from both external and internal strategy use provided unequivocal support for this hypothesis. First, concerning external strategy effects, our novel n-back strategy instruction led to improved training session performance after only a few training blocks. At posttest, compared with active controls, the strategy training group showed significantly higher accuracy on the more difficult versions of the two untrained n-back tasks but not on the other, structurally different WM tasks for which the externally provided visuospatial strategy would not be relevant. The n-back task also enabled reaction time analyses, but here the strategy training group did not differ from the controls. The present transfer pattern echoes the one typically seen after ordinary adaptive WM training that takes several weeks^8^. Second, with regard to internally generated strategy effects, both the level of detail and type of strategy (especially those similar to the externally provided one) was related to a higher performance on all three n-back tasks at posttest.

The present results using the n-back paradigm align well with previous findings mainly from complex span studies that have indicated that explicit WM strategy instruction can be effective^10–13^, and that self-generated WM strategies are associated with higher task performance^9,10,14–16^. Intriguingly, the present results also indicate that strategy instruction with just a single training session can yield a transfer pattern that mimics the one identified after ordinary 4–6-week long adaptive WM training^7,8^. As more demanding tasks are typically more sensitive to changes, it appears logical that these transfer effects following external strategy instruction emerged for the more difficult (3-back) versions of the untrained n-back tasks. While the ordinary adaptive WM training does not entail any strategy instruction, the observed relationships between internal strategy use and n-back performance strengthen the conclusion that strategies play an important role in adaptive WM training outcomes. At posttest, the majority of our controls reported use of some strategy after just the 30-minute training (the active control group) or even with no training at all (the passive control group), suggesting that strategy influences are present from the earliest stages of WM training. Interestingly, the strategy reports of our controls showed that several descriptions that were associated with higher n-back performance actually came close to the visuospatial strategy we explicitly taught to the strategy training group.

Due to the brevity of our training, we cannot draw conclusions about strategy effects in longer-term WM training, but it seems feasible to assume that several weeks of adaptive training provides ample opportunities to further develop and refine strategies for the trained tasks^9^. The main comparison in typical WM training studies is between an (uninstructed) training group and a control group, a comparison that did not show any pre-post difference in n-back performance in our short-term study. The lack of any pre-post difference between our active and passive controls is most likely due to the brevity of our training. Instead of the typical 10–15 WM training sessions over 4–6 weeks that would give ample opportunities to generate, try out, refine, and routinize strategies that would suit for the training task at hand, our active control group had only a single 30-minute session with digit n-back.

Is strategy a cause or an effect of high WM performance? With regard to the effects of external strategy instruction that we employed, strategy is clearly the cause, as the Strategy Mediation hypothesis suggests. According to this hypothesis, strategies help to make the most out of the available WM resources^10^. Following a general model of skill learning proposed by Chein and Schneider^21^, the generation and maintenance of strategies would engage metacognitive and executive functions linked to WM (e.g., the Central Executive in the multicomponent model of WM by Baddeley and Hitch^22^). However, the reverse causal claim, labeled as the Strategy-As-Effect hypothesis, has also been put forth. According to this alternative hypothesis, individuals with a higher WM capacity to start with have more cognitive resources available for generating effective strategies while performing demanding WM tasks^15^. To probe for this issue in our data, we calculated the correlation between the n-back pretest composite score and the n-back pre-post gain in the strategy training group. This correlation was negative, *r* = −0.358, *p* = 0.025, 95% CI [−0.617, −0.092], suggesting that individuals with a worse pretest WM performance benefited more from the strategy instruction. This could be taken as support for the Strategy Mediation hypothesis. However, it may be that both hypotheses are correct but that they relate to different subgroups, with Strategy Mediation working best for low WM capacity individuals and Strategy-As-Effect being relevant for persons with high WM capacity who would be able to create an effective internal strategy early on. Be it as it may, one should note that both hypotheses speak for the important role of strategies in WM performance.

In conclusion, the present results shed light on the mechanisms of WM training by indicating that participants’ strategies, be they external or internal, play an important role in WM training outcomes. This is in line with the Strategy Mediation hypothesis (strategy employment can make the use of limited WM capacity more effective) rather than the Capacity hypothesis (WM training expands WM capacity). As the present study focused on the early stages of training, future research should look into the evolvement of strategies and their relationships to WM performance measures over a longer term. If the Strategy Mediation hypothesis will gain further support, it could be time for a new strategy-based focus on memory training, bringing it back to its roots in mnemonics.

## Methods

### Participants

Finnish-speaking monolingual adults aged between 18–40 years were recruited from the University of Turku, the Turku University of Applied Sciences, Humak University of Applied Sciences, the University of Helsinki, Metropolia University of Applied Sciences, and Häme University of Applied Sciences. The enrolled participants were excluded if they had any history of psychiatric and neurological illnesses (including dyslexia), reported using CNS medication, had been exposed to two or more languages before the age of six, or had participated in previous working memory training studies. All participants were screened for depression with the Patient Health Questionnaire (PHQ-9^23^; cutoff was a total score of *≥* 9 points). The research procedures were approved by the Institutional Review Board of the Departments of Psychology and Logopedics, Åbo Akademi University, and were in accordance with the Helsinki Declaration. All participants gave their informed consent before starting the study.

Altogether 127 eligible participants took part in the pretest session and underwent block randomization into three groups. Five participants (strategy training group *n* = 1, active control group *n* = 3, passive control group *n* = 1) withdrew after the randomization. Two participants (active control group *n* = 2) had participated in previous cognitive training studies administered by our lab, and were thus excluded. One participant (strategy training group *n* = 1) was excluded because of missing training data (this participant had trained on a tablet computer which our platform does not support), and one participant (passive control group *n* = 1) was excluded because of completing the posttest a week too late. Moreover, two participants (active control group *n* = 1, passive control group *n* = 1) were excluded for being multivariate outliers at pretest (Mahalanobis distance value χ2 (14, 118) = 36.12, *p* = 0.001)^24^. After excluding the multivariate outliers, our final sample consisted of 116 participants (strategy training group *n* = 40, active control group *n* = 37, passive control group *n* = 39).

### Procedure

**Figure 4:**
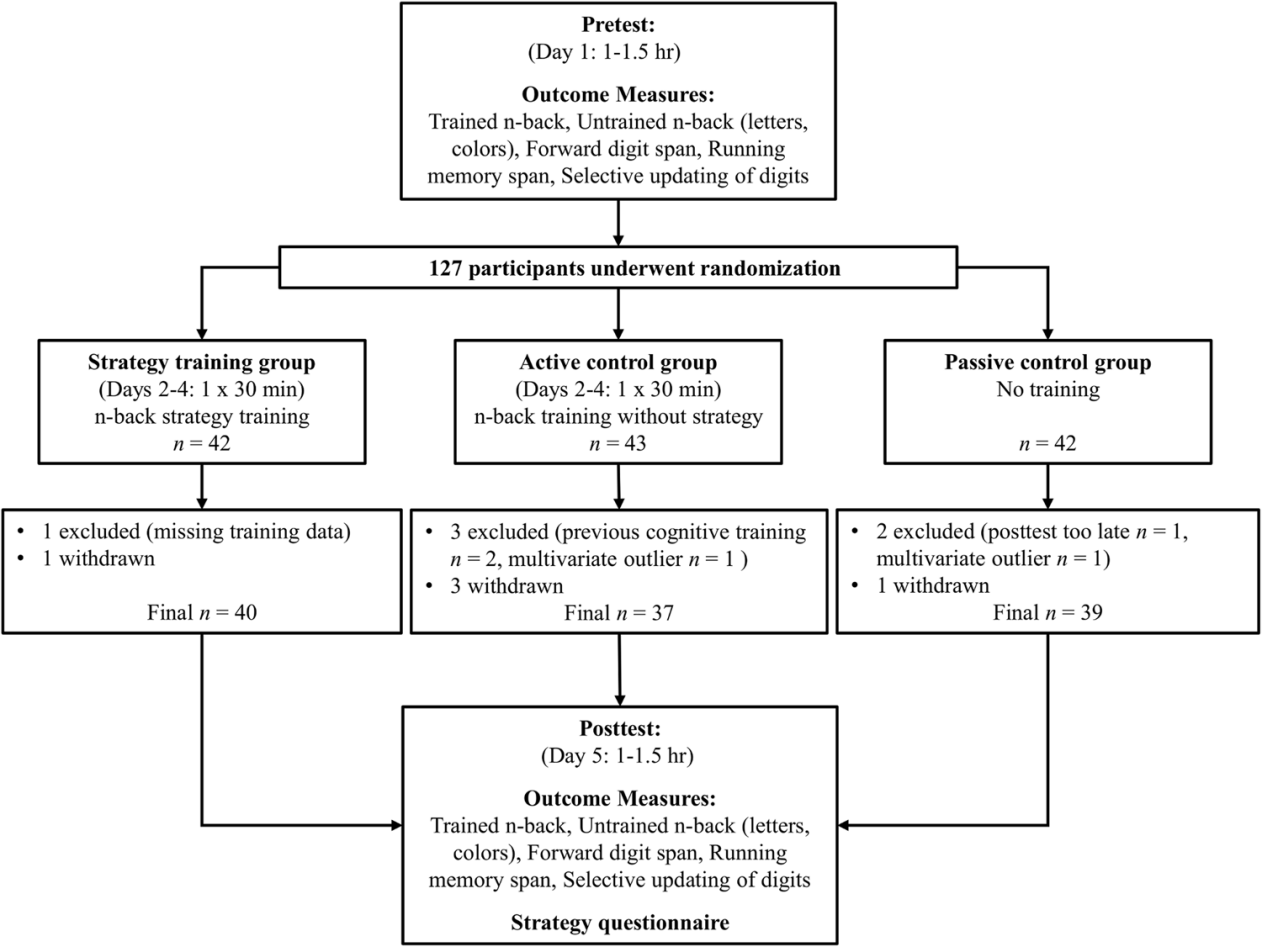
Flowchart of the study design including the attrition rate.

We employed a between-subjects pretest–posttest intervention design and randomized participants into a strategy training group, an active control group, or a passive control group (see Fig. 4). The strategy training group was given a novel visuospatial strategy for the n-back in the beginning of the training session, whereas the active control group trained on the same task the same amount of time but without any strategy instructions. The passive control group did not perform any training between the pre- and posttest. For each participant, the study was conducted within five weekdays. The pretest was completed on Monday, the training session on either Tuesday, Wednesday, or Thursday, and the posttest on Friday. The pre- and posttest sessions were run in computer classes with 1–12 participants per session, while the training session was performed online in home-based settings. Data collection took about 7 weeks, with 20 participants completing the study per week.

Prior to randomization, the participants were informed that they would be allotted either to a training group or a control group. Thus, they were not aware of the existence of two different training groups (i.e., the strategy group and the active control group). Moreover, we instructed participants not to discuss the setup during the training period with other persons participating in the study. On completion of the experiment, we also advised the participants not to reveal anything concerning their own training to persons who currently were participating in the study or who might participate in the near future.

During the experiment week, the two training intervention groups performed a single adaptive n-back training session that lasted approximately 30 minutes. Before the training started but after the strategy instruction had been given to the strategy training group, both groups filled out an expectation survey. The survey assessed the participants’ subjective expectations of training improvements (i.e., “How much do you think you will improve on the training task during this training session?”) on a 10-point Likert scale (10 = “a large improvement”, 1 = “no improvement at all”). Immediately after the training session, both groups were also asked to rate their motivation and alertness on a 1–5 scale, with 5 being most motivated/alert. Additionally, all three groups were asked to separately evaluate for each task whether they expected to perform better at posttest compared to pretest (i.e., “You completed a task called n-back with letters. How do you think you will perform in this task at posttest compared to pretest?”). This survey was administered in conjunction with the first one to the two training groups. The passive control group filled out this survey during the weekdays between the pre- and posttest. These task expectations were assessed on a 1–10 Likert scale (10 = “a much better performance compared with the pretest”, 1 = “the same performance as in the pretest”).

The pre- and posttest sessions both lasted approximately 1–1.5 hours and were identical in all but two regards: (1) the test for red-green color deficiencies was only included at pretest, and (2) at posttest an additional strategy questionnaire was implemented. The strategy questionnaire was incorporated at the end of the posttest in order to ask participants what kind of strategies they had employed in the pre- and posttest tasks. Lastly, upon the conclusion of the experiment, the existence of the three experimental groups was revealed to each participant.

### Working memory training

The n-back training task was identical for both the strategy training group and the active control group, except that the strategy training group initiated the training session by receiving the visuospatial strategy instruction (see the strategy instruction, below). In the n-back training task, digits ranging from 1 to 9 were presented for the participant one at a time on a computer screen. The participant was instructed to respond to each stimulus with either a yes or a no response on a computer keyboard. The task was to indicate if the current digit corresponded to the digit presented *n* items back in the sequence; if it did correspond one was to press a “yes” key and if not, to press “no”.

The training task comprised 20 blocks of 20 + *n* trials. The level of *n* was 1–9, starting from a 1-back sequence in the first block. The training task was adaptive, that is, the computer automatically adjusted the level of *n* after each block. If the participant responded correctly on 18–20 of the trials, *n* was increased by one. If the number of correct responses was 15–17, *n* remained the same. If more than five errors were made in a block, *n* was decreased by one. Each sequence contained 6 targets and 14 non-targets. To avoid familiarity-based responding, at least 4 out of the 14 non-targets were lures, that is, identical to the target items except that they were presented n ± 1 back (not applied in the 1-back condition).

The participants received task instructions at the beginning of each new sequence, and they were instructed to press a key in order to begin. Each sequence was structured as follows: a blank screen for 450 ms - a digit displayed for 1500 ms - another blank screen for 450 ms - the next digit. This continued until the end of the sequence, after which the instructions for the next sequence appeared. Thus, participants had altogether 1950 ms to respond to each trial. The digit sequences were randomized in each block.

#### The strategy instruction

**Figure 5:**
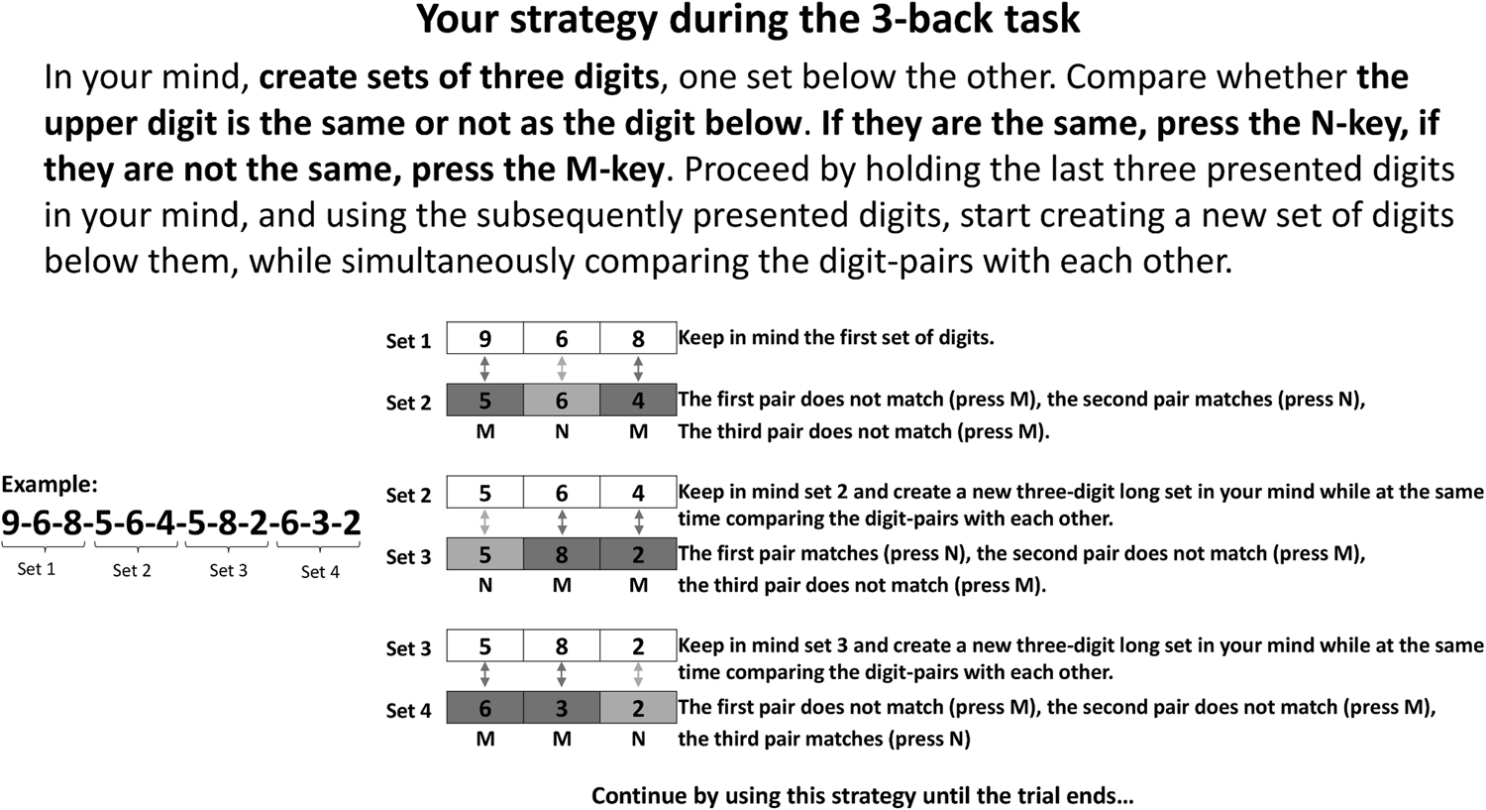
An illustration of our visuospatial strategy instruction at the 3-back level. Note that the string lengths varied as a function of the level of *n* the participant was at.

The instructions for the novel visuospatial strategy that we developed for the digit n-back task are illustrated in Fig. 5. This strategy prompted the participants to mentally order the incoming stimulus sequence into two parallel *n*-length digit strings. These two strings were to be set, digit by digit, on top of each other. This enables one to directly compare each upper and lower digit mentally as to whether they match (“yes” response) or not (“no” response). After having compared all digit pairs in the two parallel strings, the upper string is to be discarded, followed by the immediate creation of a new string underneath the lower string. The strategy instructions were presented each time a new n-back block started.

### Pre- and posttest measures

#### Trained digit n-back task

This digit n-back task was otherwise identical to the adaptive version used in training, except that the number of blocks was 10 instead of 20. The dependent variables were the maximum level of *n* achieved, and the average level of *n* achieved.

#### Task-specific near transfer measures

We measured task-specific near transfer with two untrained n-back tasks, namely an n-back task with letters, and an n-back task with colors. These non-adaptive n-back tasks contained a 2-back and a 3-back block, the order of which was randomized. Both blocks included 48 items, out of which 16 were targets and 32 were non-targets. Half of the non-targets were lures (8 *n* + 1 lures, 8 *n* − 1 lures). The sequences began with task instructions. After this, each item was shown for 1500 ms, followed by a blank screen for 450 ms. The dependent variables were accuracy as measured by d-prime^25^ and mean reaction time (RT) on correct target responses.

#### Task-general near transfer tasks

(1) Selective updating of digits (SUD) is a WM updating task based on Murty and colleagues^26^. Five digits from 1 to 9 were presented on the computer screen in a row of five boxes. The participants were instructed to memorize the digit sequence. After this, the initial digit sequence disappeared and a new row of five boxes was displayed. Two of the new boxes contained digits, while three were empty. The participants were prompted to replace the old digits with the newly presented digits in the memorized sequence, while maintaining the unchanged digits in WM. The participants completed 10 baseline trials (i.e., no updating stages), and 10 trials with three updating stages (i.e., replacement of old digits with new ones). At the end of each trial, the participants were instructed to report the final digit sequence, including the most recent updates.

When creating the SUD task digit sequences, we applied the following rules: (1) the digit updates were not allowed to occur in adjacent boxes, (2) adjacent digits in a row must deviate with more than one from each other (i.e., 4 could not be flanked by 3 or 5), and (3) the two updated digits were not identical. The order of the trials was randomized for each participant, and the participants were unaware if the next trial was an updating trial or a baseline trial. The initial digit sequence was shown for 4000 ms, followed by a 100 ms blank screen, after which the first updating stage was presented for 2000 ms. The updating stage was once again followed by a 100 ms blank screen and the next updating stage. After all the updating stages had been presented (none in the baseline condition), a recall grid with horizontally aligned boxes containing the numbers from 1 to 9 appeared on-screen. The participants were to click on the numbers in correct order. The dependent variable was the percentage of the total number of correctly recalled digits (in correct order) in the updating trials.

(2) Forward simple span. In this task, digit sequences with 4 to 10 digits (one trial per sequence length) were presented. The participants were to recall the digits in the same order they were presented. The order of the trials was randomized for each participant. Each number sequence started with a fixation cross in the middle of the screen that was shown for 500 ms, after which a digit appeared on the screen for 1000 ms. This procedure continued until the end of the sequence. At the end of each sequence, the participants were to recall the digits by clicking on the correct digits in a row of horizontally aligned boxes with the numbers from 1 to 9. The dependent variables were the total number of correctly recalled items in the correct serial position, and maximum span (i.e., highest span length where all items were recalled correctly in the correct order).

(3) Running memory. In this task, the participants were instructed to report the last 4 digits in sequences that were 4–11 items long. Altogether 8 trials were administered in random order, with one trial per sequence length. Each trial began with a fixation cross shown in the middle of the screen for 500 ms, followed by a digit for 1000 ms. This continued until the end of the sequence, where a recall grid with horizontally aligned boxes ranging from number 1 to 9 appeared, and the participants were to click on the correct digits in the right order. The dependent variable was the total number of correctly recalled items in correct position.

### The strategy questionnaire

Immediately following the posttest task battery, a questionnaire concerning the participants’ strategy use in the pre- and posttest tasks was administered. These retrospective evaluations yielded separate evaluations for each pre-posttest task. Each evaluation started with a yes/no question asking whether the participant used any strategy at pretest. Following a positive response, the participant was instructed to describe the strategy employed in a subsequent empty comment field (maximum 1000 characters). Secondly, the participant responded to a two-choice question probing a possible change in strategy use from pretest to posttest. A yes-response prompted the participant to describe the strategy change in an empty comment field.

### Statistical analyses

The background characteristics of the three groups at pretest were compared with one-way ANOVAs for continuous variables and χ^2^ tests for categorical variables. Independent samples t-tests were employed to evaluate potential differences in motivation and alertness during the intervention period between the two training groups.

As regards the pre-post analyses, ANCOVAs with posttest performance as the dependent variable, pretest performance as the covariate, and group as the between-subjects factor were conducted for each pre/post task. Significant main effects of group were followed up with planned contrasts. The first contrast compared the strategy training group to the active control group (−1, 1, 0), and the other contrast compared the strategy training group to the passive control group (−1, 0, 1). (Note that unlike in the pre-registration where we stated that post hoc tests will be used, we instead chose to employ planned contrasts. We considered a priori planned contrasts to be more adequate here, after all: we had a clear hypothesis on the strategy training effects. In practice, post hoc contrasts would yield the same results). To correct for multiple comparisons, Benjamini-Hochberg adjusted *p*-values were applied for group comparisons on each pre-post outcome measure^27^.

To examine whether the present sample had enough power to detect significant group (between-subjects) × time (within-subjects) interactions if present in the pre- and posttest measures, we conducted a post-hoc power analysis using G*Power version 3^28^. The power to detect a large (*f* = 0.40) or medium effect (*f* = 0.25) was > 0.99 based on the sample size and the use of the within-subjects correlation of *r* = 0.68 (which was the largest observed correlation across all pre-and posttest variables). We also ran a power analysis with the smallest correlation among the pre- and posttest variable (*r* = 0.32). In this case, the power to detect a large or medium effect was 0.98. For ANCOVA analyses, the power was 0.97 for detecting a large effect size (*f* = 0.40) and 0.66 for a medium effect size (*f* = 0.25). Earlier studies with WM strategy intervention have not explicitly reported results from power analyses^10–13^. Nevertheless, the two previous studies reporting effect sizes have shown large effects (${\eta }_{{p}}^{2}$ range 0.18–0.27) with groups ranging from 27 to 79 participants^12,14^. Thus, the sample size of the present study should be sufficient to achieve adequate statistical power for our strategy intervention.

The ANCOVA analyses were conducted using IBM SPSS Statistics 23 software^29^. Planned contrasts, figures, and inter-rater reliability analyses (when evaluating self-reported strategies) were performed in the R environment version 3.3.3^30^, using the packages ggplot2^31^, and rel^32^. Prior to the data collection, the study protocol was preregistered at AsPredicted.org (https://aspredicted.org/b8rp8.pdf).

### Data availability

The datasets collected and analyzed in the current study are available from the corresponding author on reasonable request.

## Electronic supplementary material

Supplementary information
