# Supplementary material for: The early effects of external and internal strategies on working memory updating training
Source: Sci Rep. 2018 Mar 6;8:4045. doi: 10.1038/s41598-018-22396-5 (PMC5840432; doi:10.1038/s41598-018-22396-5)
Supplement: Supplementary file 1 — Supplementary information [file 41598_2018_22396_MOESM1_ESM.pdf]

Supplementary information

The early effects of external and internal strategies on working memory updating training

Matti Laine[1,2*], Daniel Fellman[1], Otto Waris[1], & Thomas J. Nyman[1]

[1]Department of Psychology, Åbo Akademi University, Turku, Finland. [2]Turku Brain and Mind Center, University of Turku, Finland. Correspondence and requests for materials should be addressed to M.L. (email matlaine@abo.fi)

Supplementary Table S1 online

Classification scheme for strategy types based on participants' self-reports at posttest. The examples were created as an aid for the independent raters.

**Strategies**	**Examples**
Rehearsal	• "I repeated the digits silently in my mind"<br>• "I repeated a list of letters in my mind"
Grouping	• "I created groups of 3 digits"<br>• "I grouped the letters in pairs"
Updating	• "I created a group of digits in my mind and dropped the last digit when a new digit appeared"
Grouping and comparison	• "I split the digits into different series, and compared those to each other"
Semantics	• "I created words from the letters (e.g., C-R-S = Corn – Rose – Sand)"<br>• I converted the digits to melodies, e.g., 1356 = DO-MI-SO-LA"
Phonology	• "I made up lists based on first syllables of the digit words" [*n.b. the names for digits are multisyllabic in Finnish*]
Imagery	• "I tried to associate each digit with some image in my mind"
Familiarity	• "I chose the letters that felt most familiar"
Guessing	• "I just used intuition"<br>• "I started somewhere in the middle of the sequence, and did not memorize the first digits in the sequence at all"
Other strategies	• "I made up a song based on the letters"<br>• "I counted the digits with my fingers"
No strategy use	• "I tried to keep all the digits in my mind"<br>• "I put the [*visually presented*] digits in my visual memory"

Supplementary Table S2 online

The frequency of occurrence of reported strategy types by the control participants (active and passive controls combined) in the three n-back tasks at posttest

	Trained n-back task		n-back task with letters		n-back task with colors	
**Strategy**	*n*	%	*n*	%	*n*	%
No strategy	14	18.4	22	29.3	24	32.0
Rehearsal	18	23.7	16	21.3	25	33.3
Updating	7	9.2	7	9.3	6	8.0
Grouping	14	18.4	7	9.3	6	8.0
Grouping and comparison	16	21.1	14	18.7	9	12.0
Semantics	0	0.0	2	2.7	1	1.3
Phonology	1	1.3	2	2.7	1	1.3
Imagery	1	1.3	1	1.3	1	1.3
Familiarity	0	0.0	1	1.3	0	0.0
Guessing	1	1.3	1	1.3	1	1.3
Other strategy	4	5.3	2	2.7	1	1.3
